# Combined use of satellite and surface observations to study aerosol optical depth in different regions of China

**DOI:** 10.1038/s41598-019-42466-6

**Published:** 2019-04-16

**Authors:** Mikalai Filonchyk, Haowen Yan, Zhongrong Zhang, Shuwen Yang, Wei Li, Yanming Li

**Affiliations:** 1 Faculty of Geomatics, Lanzhou Jiaotong University, Lanzhou, 730070 China; 2 Gansu Provincial Engineering Laboratory for National Geographic State Monitoring, Lanzhou, 730070 China; 3 School of Mathematics and Physics, Lanzhou Jiaotong University, Lanzhou, 730070 China; 4 Lanzhou Petrochemical Polytechnic Information Technology and Education Center, Lanzhou, 730070 China

**Keywords:** Atmospheric chemistry, Atmospheric chemistry

## Abstract

Aerosol optical depth (AOD) is one of essential atmosphere parameters for climate change assessment as well as for total ecological situation study. This study presents long-term data (2000–2017) on time-space distribution and trends in AOD over various ecological regions of China, received from Moderate Resolution Imaging Spectroradiometer (MODIS) (combined Dark Target and Deep Blue) and Multi-angle Imaging Spectroradiometer (MISR), based on satellite Terra. Ground-based stations Aerosol Robotic Network (AERONET) were used to validate the data obtained. AOD data, obtained from two spectroradiometers, demonstrate the significant positive correlation relationships (r = 0.747), indicating that 55% of all data illustrate relationship among the parameters under study. Comparison of results, obtained with MODIS/MISR Terra and AERONET, demonstrate high relation (r = 0.869 - 0.905), while over 60% of the entire sampling fall within the range of the expected tolerance, established by MODIS and MISR over earth (±0.05 ± 0.15 × AOD_AERONET_ and 0.05 ± 0.2 × AOD_AERONET_) with root-mean-square error (RMSE) of 0.097–0.302 and 0.067–0.149, as well as low mean absolute error (MAE) of 0.068–0.18 and 0.067–0.149, respectively. The MODIS search results were overestimated for AERONET stations with an average overestimation ranging from 14 to 17%, while there was an underestimate of the search results using MISR from 8 to 22%.

## Introduction

Atmospheric aerosol is the most common in natural conditions type of disperse system, consisting of solid and liquid particles, suspended in the atmosphere. Studies of broad spectrum of atmospheric aerosol physical characteristics and chemical composition enable to anticipate potential climate changes, having dangerous and long-term ecological consequences^1–6^. Atmospheric aerosols, such as aerosols from the burning of biomass, dust minerals, volcanic ash, smoke, sea salt and particulate matter, stand out as a result of various natural and anthropogenic influences. Aerosols are important components in the Earth’s system^7^ and have a decisive influence on global and regional climate change^8–11^, air quality^10,12^, human health^13–17^, flora and fauna^14,18^ through the direct and indirect radiation forcing, and also has a direct impact on cloud processes^12,19,20^, visibility variation^21,22^. In addition, aerosols have a significant impact on the concentration, distribution and hydrological cycle of greenhouse gases, affecting the physical and chemical processes in the atmosphere.

The aerosol optical depth (AOD) is the single most comprehensive variable for the remote assessment of the aerosol load in the atmosphere and which is used to reflect aerosol column loading. Recently, satellite remote sensing and ground-based observations have become widely used to monitor the spatial and temporal distributions of aerosols on a global and local scale^23–27^. Such satellite instruments as Moderate Resolution Imaging Spectroradiometer (MODIS), Multi-angle Imaging Spectroradiometer (MISR) are used to monitor aerosols in regional and global scale and provide for long-term and continuous coverage of the territory under study. But due to the impact of surface albedo, geography and relief as well as retrieval algorithms, the precision of data reception requires continuous improvement^28–31^. Data, received from ground-based stations, has higher precision of measurement at the low spatial reach, while many China organizations and institutions created their own networks for aerosols monitoring. Such ground based optical networks for aerosols monitoring like Aerosol Robotic Network (AERONET)^29,32,33^, China Aerosol Remote Sensing Network (CARSNET)^23,34^ and Chinese Sun Hazemeter Network (CSHNET)^26,35^ use sun photometers or sun-sky radiometers.

Extensive studies concerning aerosols characteristics in China were carried out over recent years. For instance, Filonchyk *et al*.^36^ reported about statistical analysis of aerosol optic properties in the period of large dust storms in Northwest China, having revealed a clear relationship between AOD and particulate matter (PM), and about investigation of temporal and spatial AOD variations in large city districts and industrial areas of the country^37^. Ma *et al*.^38^ analyzed aerosol optical properties in mountainous areas. Xia *et al*.^27^ studied aerosols optic properties in high set areas of Tibetan Plateau and desert regions of Taklamakan Desert. The investigations covered the most rapidly developing regions of the country, such as the Yangtze River Delta^39,40^, the Pearl River Delta^41,42^, the North China Plain^20,43^ and the Sichuan Basin^44,45^. Also, to study regional and general prerequisites of atmosphere pollution, aerosol properties in many large cities of the country, including Shanghai^46^, Nanjing^47^, Beijing^48^, Guangzhou^49^, Lanzhou^37^, Tianjin^50^ and Xian^51^ have been studied. However, the majority of investigations are focused on the study of particular territories with use of specific satellite and ground instruments. Since it is difficult to establish comprehensive body of data on aerosols evolution over China due to the fact that there were few investigations focused on aerosols study, or the data is irrelevant due to remoteness of the investigations conducted^52–56^, decision was taken to carry out an advanced study with comprehensive investigation, covering satellite and ground data.

The purpose of this study is to investigate time-space distribution and AOD variation over the territory of China with use of aerosol products MODIS and MISR in the period from 2000 to 2017. In order to better understand the accuracy and reliability of the data obtained, the decision was taken to carry out cross check of MODIS and MISR products as well as their efficiency with ground based aerosol measurement data, being a reference standard for determination the efficiency of aerosols retrieval algorithm based on satellites, obtained from ground based photometric observations of AERONET. Assessment of trends in AOD changes for the last eighteen years will make it possible to evaluate the efficiency of public policy on improvement of ecological situation in the country.

## Study area and Methods

### Study area

**Figure 1:**
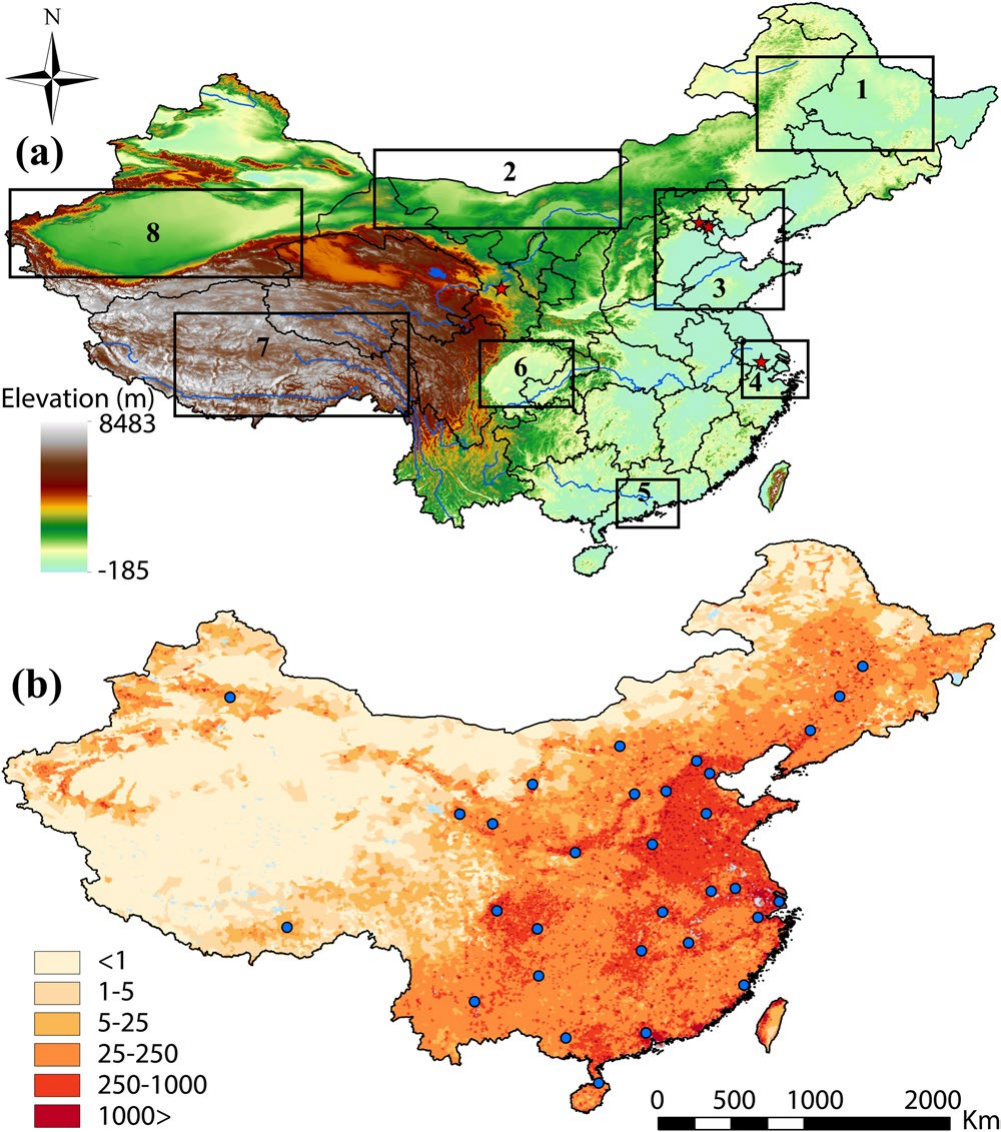
(**a**) Geographical locations of the study regions (1) Northeast China, (2) the Gobi Desert, (3) the North China Plain, (4) the Yangtze River Delta, (5) the Pearl River Delta, (6) the Sichuan Basin, (7) the Tibetan Plateau, (8) the Tarim Basin (AERONET stations are marked with asterisks) and (**b**) a map of population density of the country (people per square km).

This study does not encompass the whole territory of the country, but different ecological areas with complex and diverse relief, with desert and forest landscapes, coastal areas and hinterlands, densely-populated and underpopulated territories, which to different extents are subject to natural and anthropogenic factors of aerosols formation and effect. Thus, we have distinguished eight regions: Northeast China, the North China Plain, the Sichuan Basin, the Tarim Basin, the Tibetan Plateau, the Gobi Desert, the Yangtze River Delta and the Pearl River Delta, which are shown in Fig. 1(a).

Eastern part of China is generally more subject to anthropogenic activity, while the western China is more subject to influence of natural sources, such as dust storms. The North China Plain, the Yangtze River Delta and the Pearl River Delta are the most developed regions in China with high density of population, resulting in increase of various anthropogenic emissions. North East of China is the northernmost region of the country with abundant thick natural vegetation and is characterized by traditional sources of pollution, such as coal and oil combustion products, as well as dust, entered into the region from the deserts of north western and northern parts of China. The Sichuan Basin is surrounded by mountains, where a man-induced impact plays a key role in atmosphere pollution. The Tarim Basin and the Gobi Desert are the primary sources of dust aerosols in the process of wind erosion, and they affect both the neighboring regions, and aerosol load of areas, located away from the region of source, through advection in atmospheric circulation. The Tibetan Plateau, the most high mountain region of the country, is subject to the least anthropogenic activity due to poor population density of the territory.

### Data sources and methods

This study used spectral radiometers MODIS (Moderate Resolution Imaging Spectroradiometer) and MISR (Multi-angle Imaging Spectroradiometer), which are one of key instruments aboard of American satellite Terra series EOS, launched on December 18, 1999. Satellite Terra operates at a hight of 705 km and crosses the equator at 10:30 LST (local solar time).

MODIS has 36 spectral channels with 12-bit radiometric resolution in visible, near, middle and thermal infrared bands, and due to continuous operation and broad band of shooting (2,330 km) any territory within the station visual field is daily shot at least once. This enables to use MODIS data to perform various tasks on regular monitoring of natural phenomena within a large region. MODIS aerosol product provides daily observations of the optical depth of the aerosol (AOD) globally over the ocean and vegetation, as well as over other dark patches of earth based on Dark Target (DT) algorithm^30^ and on bright terrestrial surfaces (for example deserts) based on а Deep Blue (DB) algorithm^57^. The MODIS (Terra) datasets used in this study were downloaded at Level 1 from Atmosphere Archive and Distribution System (LAADS) Distributed Active Archive Center (DAAC) website (ladsweb.modaps.eosdis.nasa.gov) of Level-2/3 Collection 6.1 for the 18-year period from January 2000 to December 2017. With that over land MODIS AOD uncertainty is ±0.05 ± 0.15 × AOD_AERONET_^30^. As source information we used daily and averaged monthly values combined Dark Target and Deep Blue AOD at 550 nm for land and ocean with space resolution 1° and 3 km with use Scientific Data Set named “AOD_550_Dark_Target_Deep_Blue_Combined”.

Spectral radiometer MISR is a shooting system, which enables to receive the leaving Earth radiation in nine different directions. In order to thoroughly study aerosols, particles, cloud cover, water surfaces, vegetation, rocks we need some knowledge on reflected radiation in different directions. This task is performed by 9 cameras, carrying out survey in 9 different directions (nadir, 26.1, 45.6, 60.0 and 70.5°). Cameras enable to obtain images of the whole planet in four spectral bands (446, 558, 672, and 866 nm) with medium and low space resolution (from 275 to 1,100 meters). Swath constitutes 360 kilometers. Since MISR observes the same object of cloudy texture by all cameras during seven minutes, it makes possible to define wind speed. The MISR data AOD uncertainty is 0.05 ± 0.2 × AOD_AERONET_^58^. The last MISR aerosol product Level-3 (version 22) for 2018 with space resolution 0.5° and MISR aerosol product Level-2 with space resolution 4.4 km (version 23) was uploaded from Atmospheric Sciences Data Center at NASA Langley Research Center (http://eosweb.larc.nasa.gov). AOD was generated at the wave length 558 nm (Scientific Data Set named: RegBestEstimateSpectralOptDepth).

The Aerosol Robotic Network (AERONET) (http://aeronet.gsfc.nasa.gov) is one of the most commonly used networks of autoland atmospheric monitoring. It is deployed to obtain on-line large volumes of data, its accumulation and subsequent processing aimed at formation of a map of aerosol distribution over the globe. Measurements of atmospheric optical parameters are made with sun photometers CIMEL every 15 minutes in the range from 340 to 1020 nm. The total estimated uncertainty in AERONET AOD constitutes ±0.01 for longer waves (>440 nm) and ±0.02 for shorter waves^32,33^. Optical depth is calculated based on spectral attenuation of ray at each wave length with Beer-Lambert-Bouguer Law, which is based on measurement of direct solar radiation with the aim of subsequent determination of atmospheric AOD and total content of certain gases. This study used cloud-screened and quality-assured level 2.0 version 3.0 AOD product^29^. Interpolation of sun photometer AOD values at 440 and 675 to 550 nm was performed for efficient comparison with satellite data. Also for comparison with satellite data MODIS and MISR AOD average spatial values in 5 × 5 pixels around a plot of land were compared with AERONET average temporal values within ±30 minutes from satellite traveling time (at 10.30 a.m. local time).

## Results and Discussion

### Total aerosol load of territory

**Figure 2:**
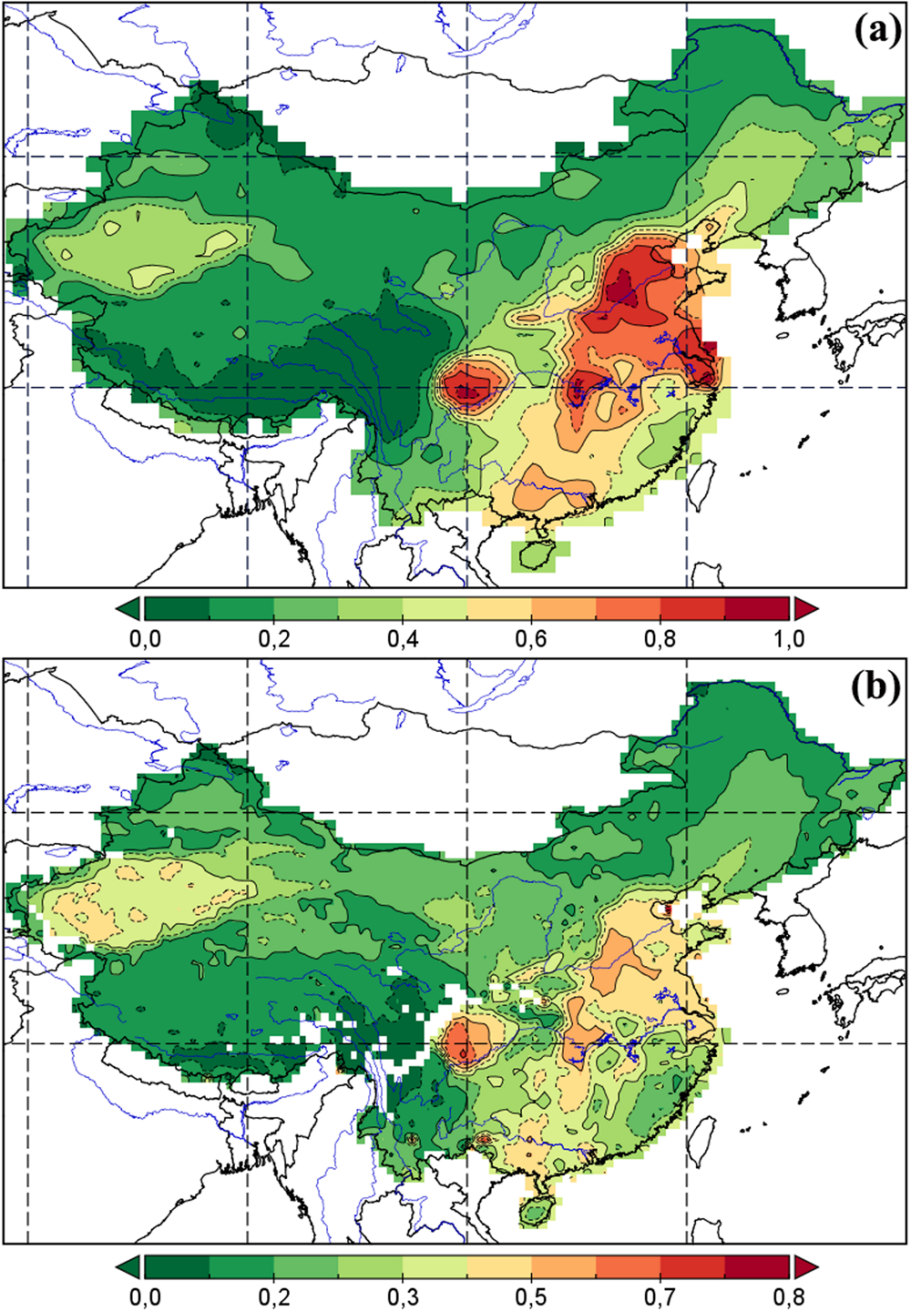
Annual average (**a**) MODIS AOD and (**b**) MISR AOD over China from 2000 to 2017.

Aerosol optical depth is one of the main parameters to determine aerosol load on atmosphere and is calculated by integrated direct solar radiation measurement data. 18-years average MODIS AOD values over the territory of China demonstrate clear spatial distribution with high values in the east of the country and with gradual decrease to the west (Fig. (2a)). In particular, three regions of east coast (the North China Plain, the Yangtze River Delta, the Pearl River Delta) and one region of the central part of China (the Sichuan Basin) are characterized by relatively high MODIS AOD annual values (over 0.6) (Table 1). There is a clear relationship between the density of population and aerosol concentration in different regions of the country. It is attributed to the fact that over 70% of the country population live in the eastern part of China^24^.

**Table 1:** Annual and seasonal means and standard deviations of MODIS AOD (550 nm) and MISR AOD (558 nm) in different regions of China for the period 2000–2017.

	MODIS					MISR				
	Annual mean	Winter	Spring	Summer	Autumn	Annual mean	Winter	Spring	Summer	Autumn
Northeast China	0.274 ± 0.147	0.233 ± 0.13	0.378 ± 0.174	0.289 ± 0.116	0.195 ± 0.086	0.25 ± 0.08	0.212 ± 0.03	0.349 ± 0.088	0.278 ± 0.073	0.179 ± 0.026
Gobi Desert	0.182 ± 0.072	0.146 ± 0.044	0.257 ± 0.06	0.206 ± 0.057	0.119 ± 0.024	0.188 ± 0.06	0.135 ± 0.031	0.267 ± 0.038	0.216 ± 0.037	0.136 ± 0.028
North China Plain	0.675 ± 0.211	0.554 ± 0.179	0.685 ± 0.116	0.915 ± 0.178	0.546 ± 0.115	0.65 ± 0.17	0.521 ± 0.078	0.66 ± 0.084	0.917 ± 0.161	0.529 ± 0.112
Yangtze River Delta	0.727 ± 0.161	0.695 ± 0.115	0.811 ± 0.1	0.788 ± 0.22	0.613 ± 0.093	0.702 ± 0.122	0.661 ± 0.079	0.79 ± 0.089	0.765 ± 0.159	0.593 ± 0.103
Pearl River Delta	0.546 ± 0.195	0.489 ± 0.102	0.75 ± 0.224	0.453 ± 0.14	0.528 ± 0.147	0.524 ± 0.149	0.452 ± 0.101	0.709 ± 0.183	0.438 ± 0.134	0.499 ± 0.116
Sichuan Basin	0.601 ± 0.162	0.422 ± 0.156	0.644 ± 0.119	0.569 ± 0.146	0.443 ± 0.124	0.582 ± 0.147	0.407 ± 0.143	0.622 ± 0.14	0.541 ± 0.139	0.429 ± 0.141
Tibetan Plateau	0.143 ± 0.053	0.1 ± 0.03	0.206 ± 0.039	0.16 ± 0.036	0.107 ± 0.015	0.135 ± 0.066	0.115 ± 0.023	0.199 ± 0.045	0.129 ± 0.032	0.099 ± 0.026
Tarim Basin	0.316 ± 0.203	0.186 ± 0.098	0.592 ± 0.176	0.311 ± 0.074	0.174 ± 0.076	0.344 ± 0.103	0.203 ± 0.059	0.63 ± 0.045	0.348 ± 0.047	0.195 ± 0.054
China	0.346 ± 0.094	0.287 ± 0.061	0.445 ± 0.071	0.382 ± 0.067	0.269 ± 0.04	0.332 ± 0.069	0.266 ± 0.037	0.441 ± 0.035	0.361 ± 0.045	0.259 ± 0.038

The North China Plain, the Yangtze River Delta, the Pearl River Delta and the Sichuan Basin are the most polluted regions of the country. They characterized by rapidly growing economies with the largest urban and industrial agglomerations and the highest density of population (Fig. 1(b)), with a great amount of emissions from industrial and agricultural activity as well as daily living needs, resulting in high aerosol load on these territories. In the Pearl River Delta density of population constitutes over 1,044 people per km^2^ and the highest density in the mainland of this region is found in Shenzhen (5,962 people per km^2^), Dongguan (3,358 people per km^2^), Foshan (1,965 people per km^2^) and Guangzhou (1,889 people per km^2^)^59^. In the Yangtze River Delta density of population is also high. It includes city of central subordination – Shanghai, one of the largest cities not only in China, but in the whole world with density of population over 3,816 people per km^2^^ 60^, and other largest cities of Jiangsu and Zhejiang provinces with an average density of population over 746 people per km^2^^ 61^. Industrialization and urbanization process, which for the last thirty years has been peculiar to all territory of the country, is characterized by consumption of enormous amount of fossil fuel (coal, oil), which results in emission of a significant amount of anthropogenic secondary aerosols^62–64^. Together with emission of anthropogenic aerosol, high MODIS AOD in the Sichuan Basin are also caused by unfavorable conditions of aerosol diffusion due to the basin orography, low wind speed, resulting in long particles residence in atmosphere, which does not contribute to aerosol diffusion for their sedimentation^22^.

The lowest MODIS AOD values are on the Tibetan Plateau, located near the Sichuan Basin. Here MODIS AOD values range from 0.025 to 0.223 over the entire territory of the region. This tremendous difference in aerosol load among the regions is associated with differences in population as well as relief. Because of the fact that a great part of the Tibetan Plateau is located at a hight over 4,000 meters, this prevents aerosol penetration from more polluted regions of the country^27,65^. Some of the lowest MODIS AOD values (from 0.143 to 0.346) were also found in regions of thick natural forest vegetation cover of Northeast China. Thus, thick vegetation, mountainous areas with low density of population and lack of human activity as well as restriction in coarse particles entering due to orography impede aerosol formation.

Desert regions the Tarim Basin and the Gobi Desert, characterized by local dust emissions, demonstrate different MODIS AOD values. The Tarim Basin is characterized by high concentrations of natural aerosols with prevalence of desert dust, emitted by the Taklamakan Desert with MODIS AOD values between 0.323 and 0.59. The Gobi Desert, the place of large deserts location, is characterized by less variations of MODIS AOD values (from 0.11 to 0.301). Since these regions are underpopulated and restricted in emissions of industrial aerosols, aerosol loads remain low for the whole year, except for spring.

**Figure 3:**
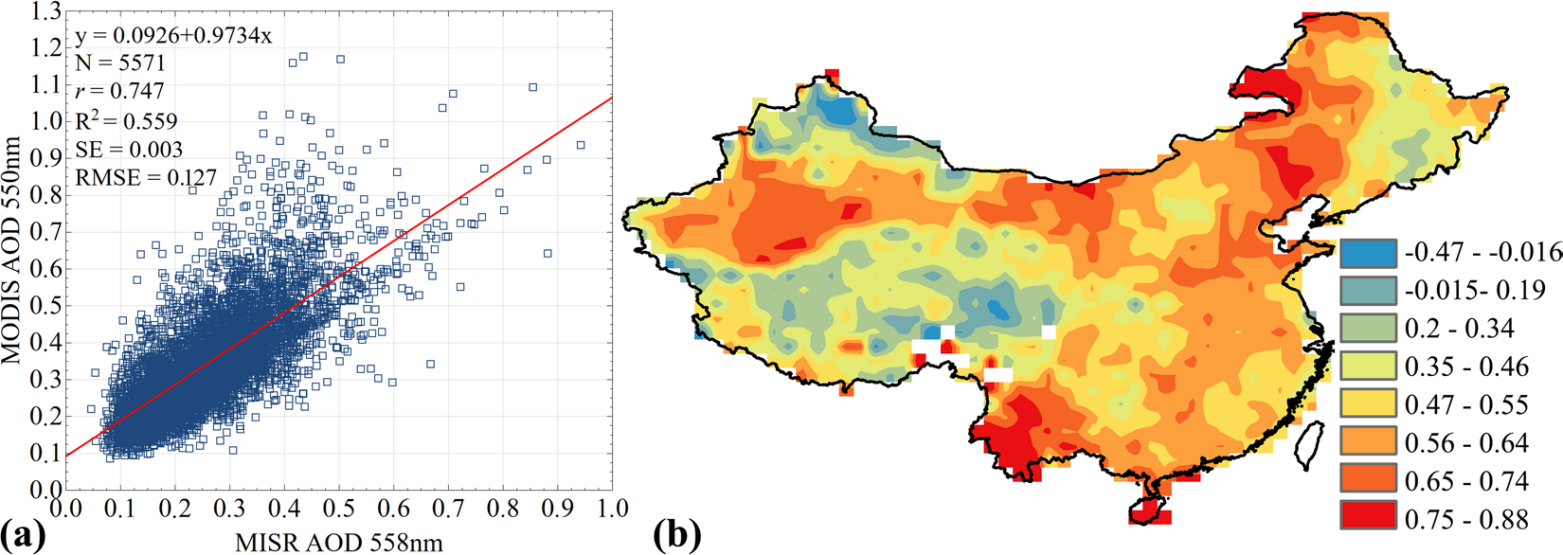
Inter-comparison (**a**) and spatial correlations (**b**) between MODIS (550 nm) and MISR (558 nm) AOD over China during 2000–2017.

AOD results, obtained with spectral radiometer MISR, demonstrate the similar results with MODIS AOD. MISR AOD data also demonstrate high AOD values in the east of the country (the North China Plain, the Yangtze River Delta, the Pearl River Delta), in the Sichuan Basin, where values vary from 0.521 to 0.828. Low values were found over the Tibetan Plateau (from 0.025 to 0.19) and in Northeast China (from 0.1 to 0.256) (Table 1 and Fig. (2b)). To study a relationship between the respective AOD statistical data, obtained with two instruments MODIS (y-axis) and MISR (x-axis), we performed a linear regression analysis, demonstrated in Fig. 3(a). “Merged product” (combined MODIS Dark Target and Deep Blue AOD), used in this study, demonstrated a relatively high correlation with MISR AOD (*r* = 0.747). Determination coefficient (R^2^) also demonstrates high values, suggesting that the model calculated parameters by 55.9% account for the dependency between the studied parameters, obtained with the two instruments. This high correlation is attributed to the fact that AOD data from the two instruments was obtained at the same time, also MODIS AOD uses the power of each of the two retrieval algorithms, which significantly decreases the share of pixels without data and thus increases a spatial coverage of the territory, catching different types of surface.

In order to obtain a more profound understanding of the chosen algorithms efficiency and reveal their values, fields of similarities and differences, correlations and differences in AOD, we analyzed a spatial correlation in the period from 2000 to 2017 (Fig. (3b)) with quantitative sampling (N) of 5,571 pairs. Since the global MISR product has a higher resolution (0.5 × 0.5°) as compared to MODIS (1 × 1°), we performed a conversion of MODIS space resolution to MISR values with use of replicate sample of the nearest neighbors. Every pixel in MODIS was divided in 4 identical pixels of 0.5 × 0.5° in size, followed by calculation of correlation coefficient for each pixel. It was revealed that medium (0.5–0.7) and high (over 0.7) correlations were in the majority of regions under study, demonstrating that the two instruments have the similar AOD retrieval algorithms. Low (less than 0.5) correlation was registered over the territory of the Tibetan Plateau, which is characterized by a high reflecting power of surface as well as absence of AOD data over the relevant areas. However, most regions with low correlation can be either desert areas with very bright surfaces or areas with complex surface elements, which leads to lower correlations between two aerosol products due to their lower sensitivity to aerosol properties on bright surfaces^28,58,66^. In general, areas with large differences between spectrometers can be divided into complex surface conditions (transition zones from bare earth to areas with dense or rare vegetation cover), complex aerosol types (inaccurate representations of aerosol microphysics in search processes on dark surfaces or dark vegetation) as well as desert areas with very bright surfaces. Also, discrepancies between MODIS and MISR data can be explained by different processing methods and different sensor calibration algorithms^24,57,67^. Nevertheless, AOD data, obtained with different instruments, were similar in the majority of the areas under study. Results of regression showed that satellite products often correlate well with one another, but struggle from slope or Y-intercept biases.

### Comparison of AOD from MODIS, MISR and AERONET

Joint analysis of results of ground and satellite measurements naturally poses a question of their faithfulness and compatibility. For the purpose of AOD data validation in the period from 2002 to 2017 we carried out the comparison of results, obtained from MODIS and MISR, with results from four stations of AERONET network (Beijing: E116°381′ N39°997′, XiangHe: E116°962′ N39°754′, Taihu: E120°215′ N31°421′ and SACOL: E104°17′ N35°946′). The tolerance of AOD observations from automatic sun photometers of global network AERONET in the visible range of solar spectrum does not exceed 0.01 (subject to the wave length more than 440 nm)^32^, and the observations of these apparatuses were taken as reference ones. The selection of these stations is determined by use of data, obtained over different types of underlying terrain, being the major uncertainty source of satellite aerosol retrievals^31,50^, and only these stations have long-term AOD data for over four years, in contrast to other AERONET stations, about 60 in China, the majority of which have short-term measurements only. However, AERONET networks data is found to be insufficient to ensure reliable AOD reconstruction by satellite data for specific areas by virtue of high rareness of stations around the territory of China. The most reliable way to obtain information about AOD consists in combined use of satellite and ground observations. Data from actinometric observations, performed by AERONET network, may be involved to obtain additional regular information about spatial-temporal variation of AOD. Such information is necessary, first of all, to advance optical models of optically active atmospheric components and improve calculations reliability.

Efficiency of aerosol extraction algorithm may be evaluated by the obtained statistical parameters of linear regression: intercept, slope, *r* (correlation coefficient), SE (standard error), RMB (relative mean bias) with a value of more than 1 or less than 1 indicate overestimate or understatement of search results of AOD, MAE (mean absolute error), RMSE (root-mean-square error), R^2^ (determination coefficient). For example, non-zero intercept indicates that at low AOD values retrieval algorithm is biased, which may be associated with sensor calibration error or mistaken assumption about surface reflection. Slope, which is greater or less than unity, indicates the likelihood of some inconsistency between aerosol models, used in retrieval algorithm, and a real model^68^.

**Figure 4:**
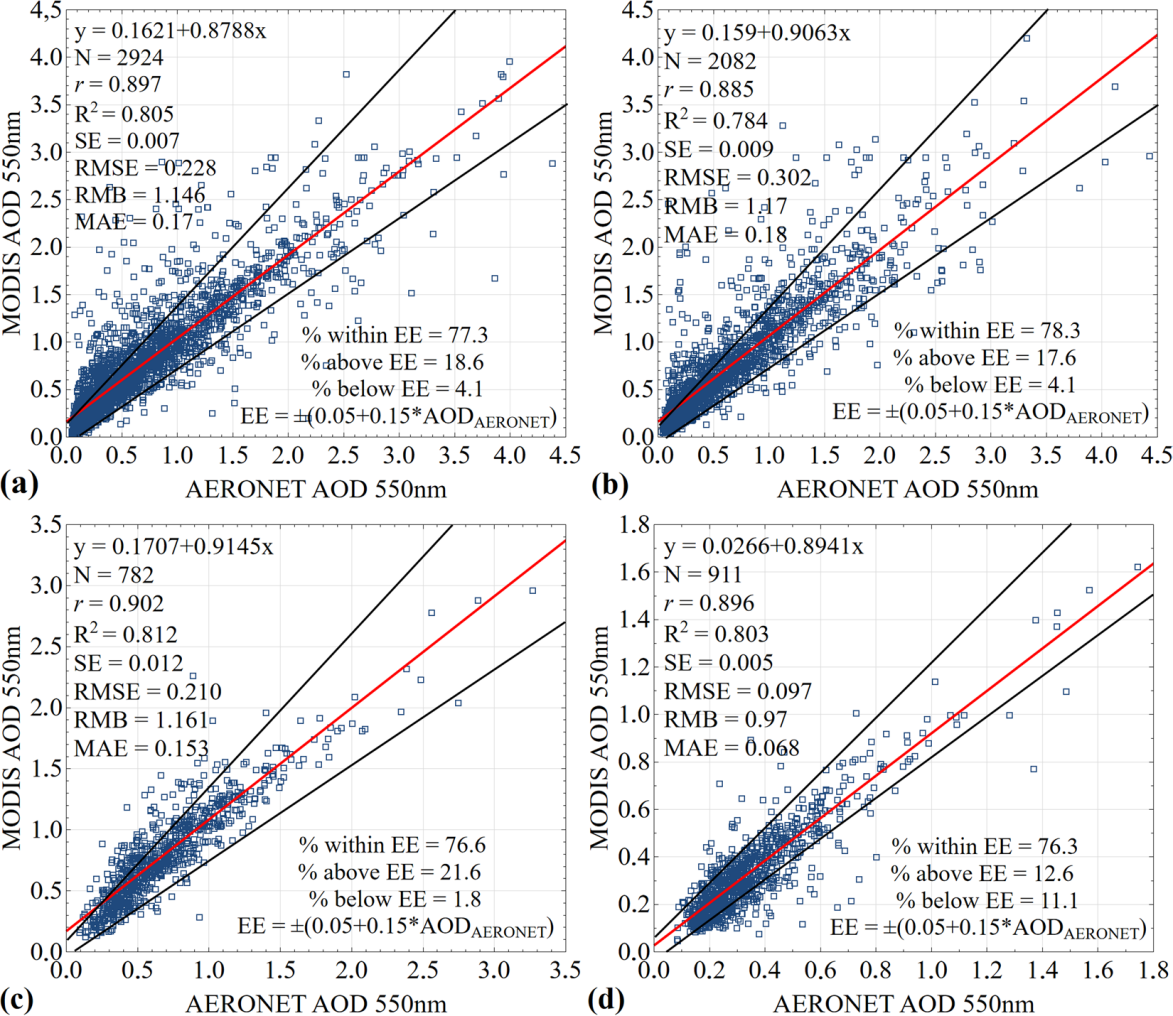
Scatter plots representing validation of MODIS AOD against AERONET AOD retrieved at 550 nm over (**a**) Beijing (2002–2017), (**b**) XiangHe (2007–2017), (**c**) Taihu (2005–2016) and (**d**) SACOL (2006–2012) stations. The red line is the regression line, and the black lines define the envelope of expected error (EE). Where N - number of matched data points, *r* - correlation coefficient, R^2^ - coefficient of determination, SE - standard error, RMB - relative mean bias, MAE - mean absolute error, RMSE - root-mean-square error at 95% confidence interval.

The results of regression and statistical measures between daily averaged data of MODIS AOD (y-axis) and AERONET AOD (x-axis), obtained at a wavelength 550 nm, are shown in Fig. 4. Notwithstanding the difference of spatial scales of ground and satellite data, intensification of multiple scattering processes under the conditions of smoke coverage, time series of AOD observations, obtained with MODIS and AERONET, comply with each other. Generally, over 76% of concerned sets of daily averaged MODIS AOD data, extracted over four AERONET stations, fall within the expected uncertainty of ±0.05 ± 0.15 × AOD_AERONET_. Best AOD results were observed at Beijing (data points of 2,924) and XiangHe (data points of 2,082) stations (Figure 4(а,b)), where 77.3% (2,260 pairs) and 78.3% (1,630 pairs) of retrieval results are within the expected error (EE), and cross-correlation coefficients between the data demonstrate high correlation (*r* = 0.897 and *r* = 0.885) subject to RMSE = 0.228 and 0.302 with low MAE = 0.17 and 0.18 overestimate search results with RMB = 1.146 and 1.17, respectively. Similar results may be attributed to the fact that the both AERONET stations are located in one geographical area and are exposed to the same sources of aerosol load. As is seen from Fig. 4(с,d), differences between MODIS and AERONET AOD are mainly fall within theoretical estimate of measurement error for Taihu (data points of 782) and SACOL (data points of 911) stations as well, demonstrating 76.6% (599 pairs) and 76.3% (695 pairs) subject to high degree of correlation between satellite and ground data (*r* = 0.902 and *r* = 0.896) and RMSE = 0.210 and 0.097 with low MAE = 0.153 and 0.068, and also RMB = 1.161 and 0.97, respectively. It was found out that MODIS has high bias against AERONET (the slope from 0.87 to 0.91 and intercept from 0.026 to 0.17), showing scatter at high correlation coefficients over different AERONET stations. With that the large intercept value (over 0.1) may be associated with uncertainty in the assessment of reflecting power of underlying terrain, indicating that urban (Beijing), suburban (XiangHe) and aquatic (Taihu) landscapes may be underestimated by MODIS retrieval algorithm. Bias and scatter suggest that compliance between AERONET and MODIS may depend on certain factors, which are not fully considered during retrieval^69^. Thus, the use of values of optical depth, regenerated based on combined algorithm (combined Dark Target and Deep Blue) from Collection 6.1, results in improvement of correlation between satellite and ground data. At the same time, it is obvious that satellite measurements of MODIS AOD over various regions showed good results, delivering quite precise values of optical depth under conditions of most typical atmospheric aerosol loads, making it possible to carry out the analysis of trend constituents of multiannual time series.

**Figure 5:**
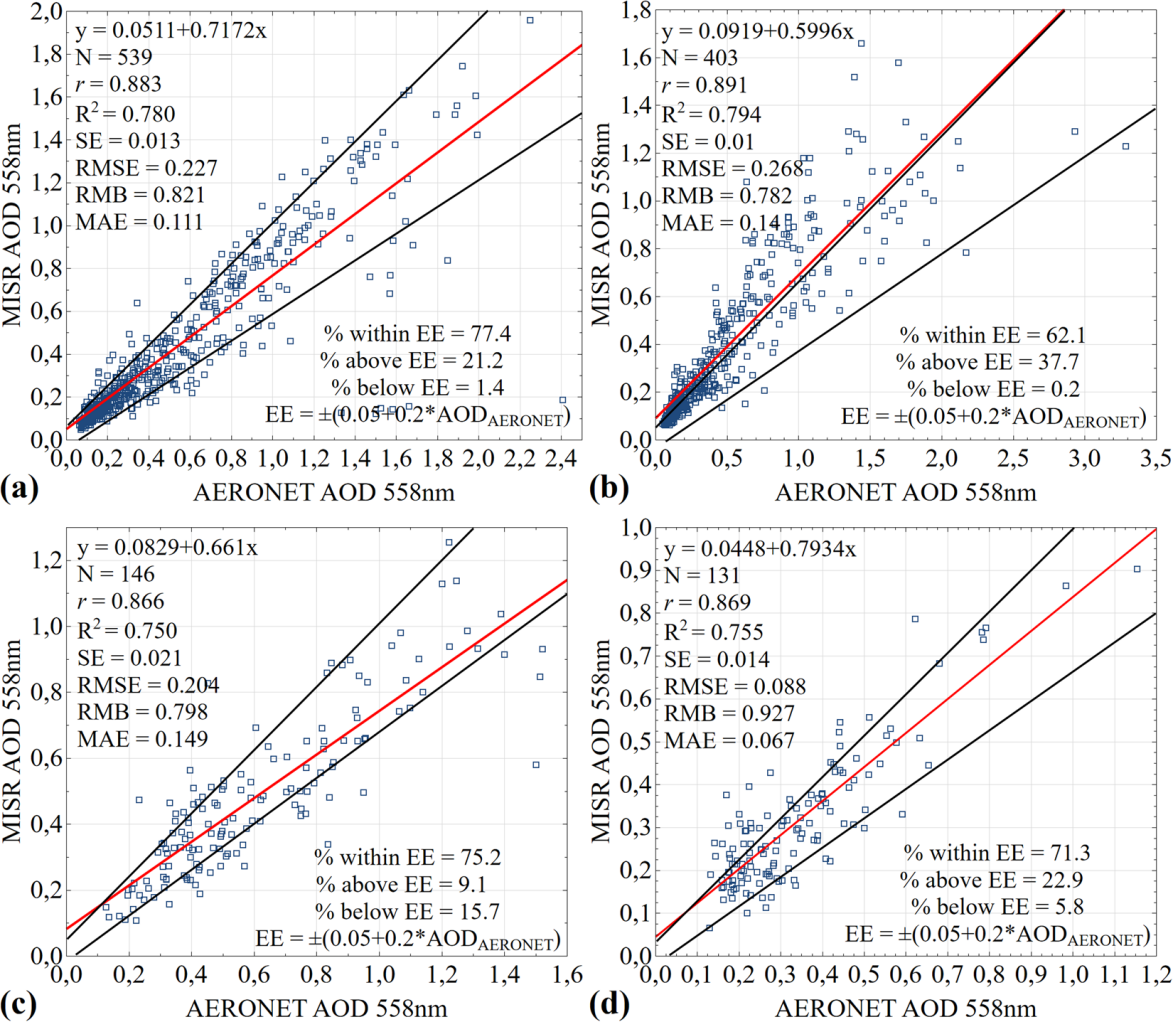
Scatter plots representing validation of MISR AOD against AERONET AOD retrieved at 558 nm over (**a**) Beijing (2002–2017), (**b**) XiangHe (2007–2017), (**c**) Taihu (2005–2016) and (**d**) SACOL (2006–2012) stations. The red line is the regression line, and the black lines define the envelope of expected error (EE). Where N - number of matched data points, *r* - correlation coefficient, R^2^ - coefficient of determination, SE - standard error, RMB - relative mean bias, MAE - mean absolute error, RMSE - root-mean-square error at 95% confidence interval.

Comparison among daily averaged AOD data, obtained with MISR (y-axis) and AERONET (x-axis) at the wavelength 558 nm in four places, is demonstrated in Fig. 5. Notwithstanding the lower numbers of pairs (N) for comparison due to less repetition frequency, MISR AOD data also demonstrate good productivity. Summary statistics shows underestimation of MISR AOD against AERONET, especially at high AOD values. It may be caused by MISR aerosol retrieval algorithm weaknesses or other factors, such as temporal and spatial aerosols variability. As is seen from Fig. 5, differences are mainly fall within theoretical estimate of AOD measurement error with use of MISR, which constitutes ±0.05 ± 0.2 × AOD_AERONET_. Generally, the results obtained at the four stations show similar regression trends with R^2^ values ranging from 0.750 to 0.794. However, the highest values were found at Beijing (data points of 539) and Taihu (data points of 146) stations (Fig. 5(a,c)), where 77.4% (417 pairs) and 75.2% (109 pairs) of retrieval results fell within the EE and demonstrated high correlation values between data pairs of MISR and AERONET, and constituted *r* = 0.883 and 0.866, at RMSE = 0.227 and 0.204 with low MAE = 0.111 and 0.149, as well as understating search results RMB = 0.821 and 0.798, respectively. MISR also operates very well when retrieving AOD in XiangHe (data points of 403) and SACOL (data points of 131) (Fig. 5(b,d)), showing a high degree of correlation (*r* = 0.891 and 0.869). With that 62.1% and 71.3% of MISR AOD fall within the expected uncertainty, at RMSE = 0.268 and 0.088 with low MAE = 0.14 and 0.067, however, the search results were understated with RMB = 0.782 and 0.927, respectively. At high correlation relationship between MISR and AERONET we may also observe a high bias (the slope from 0.59 to 0.79 and intercept from 0.044 to 0.091), demonstrating a considerable scatter. Such good results may be characterized by unique features in MISR instrument, which make it possible to ensure a better view and study of spectral response characteristics to obtain aerosol optical properties over various surfaces due to the use of its polygonal and multispectral view capabilities.

Results, which do not fall within the expected tolerance of MODIS and MISR, indicate that MODIS and MISR AOD, probably, may not be well calibrated in the heavily polluted regions with high AOD values. Although the bias falls within the expected uncertainty of MODIS and MISR retrieval algorithms, in order to obtain more precise and faithful information these algorithms need to be improved. With that the quality of aerosols retrieve and its bias depend on a number of factors, including types of surfaces, soils, aerosols, clouds reflecting power and underlying terrain. And an error in these factors may cause overestimating and underestimating of the retrieval results^69,70^. The previous investigations, aimed at the study of aerosols properties over various China cities, demonstrate different results, where in the period 2005–2011 70.5% of MODIS AOD data (Сollection 6.0) falls within the expected uncertainty at Xianghe and demonstrate high correlation (*r* = 0.91)^48^. In SACOL station in the period 2006–2012 at *r* = 0.66 only 54.52% of MODIS AOD data (Collection 005) falls within the expected uncertainty^71^. However, in the period 2002–2004 at Beijing station at high correlation (*r* = 0.93) approximately 56% of the MISR AOD (version 15) values fall within the expected uncertainty^72^. The lower compatibility indices of MODIS and MISR AOD against AERONET were at the older versions of aerosol products, but gradual improvement of calibration processes and, as a consequence, retrieval algorithms, results in the improvement of interconnection between satellite and sun photometers data over various types of underlying terrain.

### Interannual variability of AOD

**Figure 6:**
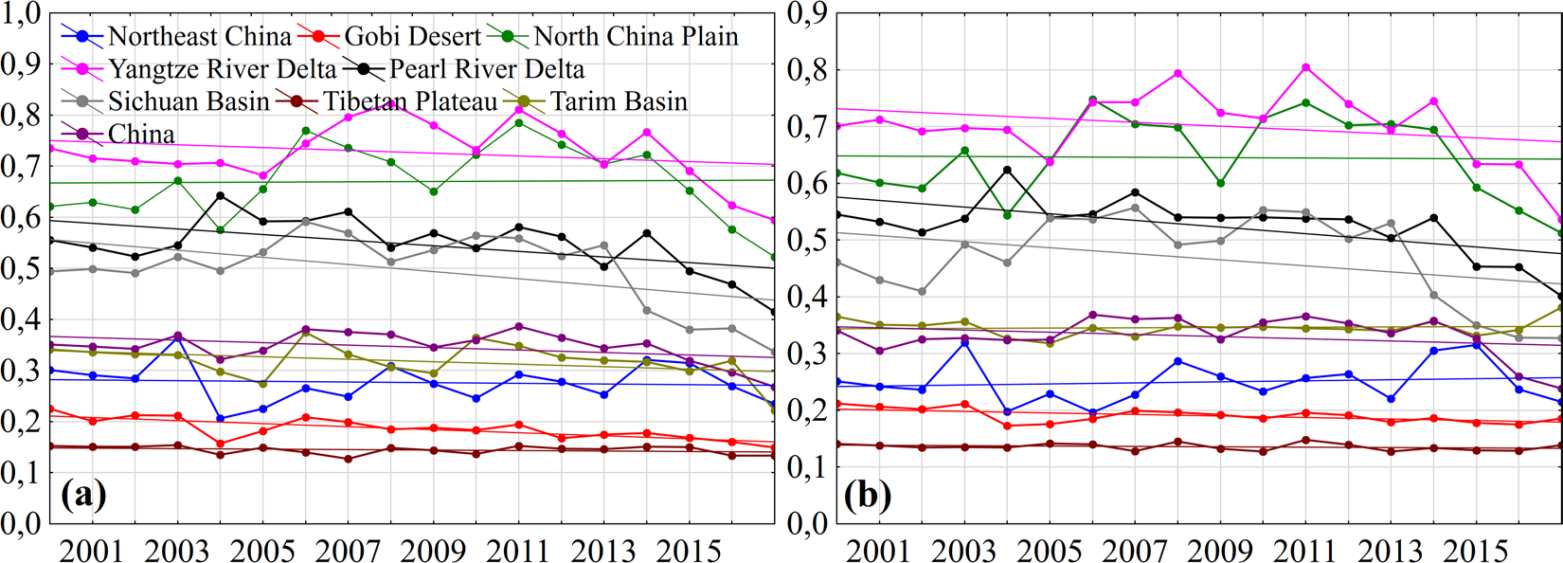
The interannual variations of the yearly mean MODIS (**a**) and MISR (**b**) AODs at different regions of the country from 2000 to 2017.

It is generally recognized that population growth, rapid pace of urbanization, economic and industrialization development contribute to increase of aerosol concentration in atmosphere. This trend is also typical for all territory of mainland China. Time series of interannual variability of MODIS and MISR AOD average values for eight regions in the period 2000–2017 are demonstrated in Fig. 6. Linear regression of MODIS/MISR AOD annual values (Figure 6(а,b)) is depicted as an unbroken line for each of the studied regions of the country. It is evident from the figure that in all geographical regions and generally in the country there is a tendency towards gradual decline in aerosol concentration to −0.004 per decade (from 0.351 in 2000 to 0.268 in 2017) for MODIS and to −0.009 (from 0.341 in 2000 to 0.238 in 2017) for MISR, where 18-years AOD values decreased by 23.6% and 30.1% on the average in the country, respectively. However, for all regions, excluding the Gobi Desert and the Tibetan Plateau, graphs have a saw tooth pattern with alteration of high and low annual mean values. The highest interannual AOD values were registered in the North China Plain and the Yangtze River Delta, where annual MODIS/MISR AOD values ranged from 0.522/0.512 (in 2017) to 0.785/0.742 (in 2011) and from 0.595/0.537 (in 2017) to 0.823/0.784 (in 2008), with gradual decline in 2017 with a trend towards −0.002/−0.006 and −0.004/−0.008 per decade (confidence level 95%), having 18-years average values 0.675/0.65 and 0.727/0.702, respectively. A trend towards gradual decline of MODIS/MISR AOD values on the average by −0.006/−0.007 and −0.009/−0.008 per decade at average values 0.546/0.524 and 0.601/0.582 is characteristic for the Pearl River Delta and the Sichuan Basin. AOD values over the Tibetan Plateau and the Gobi Desert with its gradual decline by −0.001/−0.001 and −0.004/−0.002 per decade at average values 0.144/0.135 and 0.183/0.188 changed little, if at all. Variabilities of values were irrelevant, ranging from 0.127/0.128 (in 2007) to 0.154 (in 2003 for MODIS) and 0.148 (in 2011 for MISR) over the Tibetan Plateau, and from 0.149 (in 2017 for MODIS) and 0.175 (in 2016 for MISR) to 0.213 (in 2002 for MODIS) and 0.211 (in 2003 for MISR) over the Gobi Desert. In the area with natural vegetation (Northeast China) and desert landscape (Tarim Basin) 18-years average values constituted 0.274/0.25 and 0.316/0.344, with gradual decline of −0.003/−0.005 and −0.006/−0.003 per decade. Based on MODIS and MISR data it is evident that AOD values gradually grow during 2002–2011 in all regions of the country, but from 2012 to 2017 we see their clear decline. This may be associated with strict state policy, aimed at control and reduction of amount of emissions from all anthropogenic activities.

Years long AOD tendency may depend on such factors as Asian dust storms in spring, period of biomass combustion in autumn as well as complicated cloud cover conditions^31,73–75^, thus in order to study the trends more thoroughly, it is necessary to exclude these seasonal fluctuations. May and June 2003 were excluded from deseasonalized analysis, because they were the period of active dust storms, affected the whole Asian continent^13,36,44,66^. In order to avoid potential impact of cloud fractions on AOD retrieval, data, obtained with the share of cloud fractions over 80%, were also excluded from the study^31,74^, because the results may be overestimated^24^. Seasonal anomalies were calculated, in order to exclude seasonality from data dealing with long-term trends calculation. The deseasonalized monthly anomaly is derived by subtracting the monthly average computed for the study period from a given monthly mean value. Also we used a method of moving average (doxcar method), which applied to calculate average monthly anomalies according to MODIS and MISR data by averaging out all monthly anomalies during the established time interval (set to ±11 months, that is each point ±5 months). This method is commonly used with time series data to smooth short-term fluctuations and identify main trends and cycles. It is also efficient to detect medium signals, when suppressing high-frequency data variations^31,50,74^.

**Figure 7:**
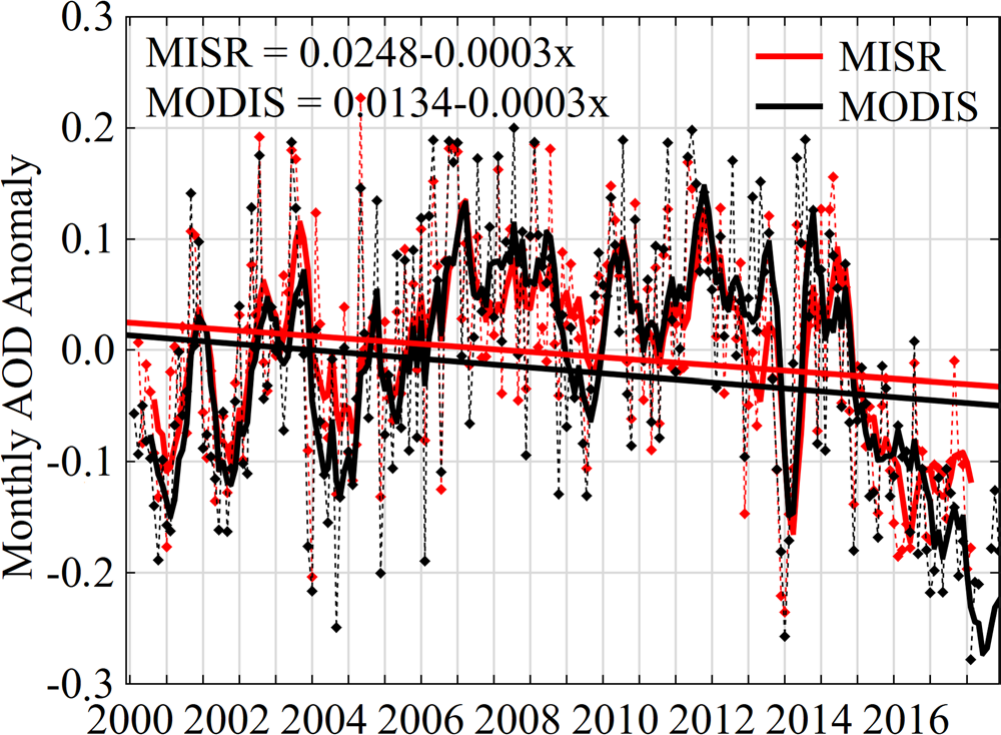
18-years deseasonalized series of Terra MODIS and MISR over the territory of China. Dashed lines demonstrate peaks of seasonality, and thick solid lines show doxcar analysis (window size, ±5 months) for MODIS (black) and MISR (red).

Figure 7 demonstrates anomalies in monthly course of AOD values. Five-month moving average helped to narrow the coverage of time series levels and therefore it more precisely reflects seasonal trends. Notwithstanding different observation conditions between MODIS and MISR, sensors demonstrate similar temporal regularities in anomalies. Anomalies for all territory of China were well correlated (*r* = 0.781) with gradual increase by 15% from 2000 to 2007 and decline by 29% from 2008 to 2017. However, even after removal all data on large sandstorms from deseasonalized analysis, the highest peaks of seasonality are observed in spring, and the lowest - in winter. As a rule, monthly standard deviation (SD) in anomalies constitutes 0.098 at SE = 0.0092 for MODIS Terra and SD = 0.099 at SE = 0.0098 (confidence level 95%) for MISR Terra. Generally, it is observed a gradual reduction of anomalies in the whole study territory.

It is evident from Figs 6 and 7 that variation in AOD, obtained from MODIS and MISR, is more or less similar to minor higher values, noticed in the data of one of instruments. However, aerosol measures and their trends, obtained from different spectral radiometer, may depend on such factors as methods of processing, calibration and retrieval algorithms^30,55,74^. Other important factors, affecting data generation, are meteorological parameters^12,45,53^, underlying terrain^26,40,43,48^, cloud cover^31,73,74^, various anthropogenic effects (biomass combustion, industrial emissions, construction activities, and etc.)^41,46,49,72,76^, as well as sensors sensitivity to differences in vegetation cover^26,38,43^.

### Seasonal and monthly AOD changes

We may note that AOD changes demonstrated not only different regional characteristics, but also various indices in one studied region, indicating high share of local sources of pollution. Thus, 18-years average MODIS AOD values over eight regions of China are characterized by high indices from the highest in Yangtze River Delta (Jinan: 0.963, Shanghai: 0.886, Hangzhou: 0.863), the North China Plain (Beijing: 0.856 and Tianjin 0.848), the Sichuan Basin (Chengdu: 0.890, Chongqing: 0.799), the Pearl River Delta (Guangzhou: 0.646), Northeast China (Harbin: 0.447) to the lowest over the Tibetan Plateau (Lhasa: 0.111). The fact that the lowest AOD are found in the region of the Tibetan Plateau, and some of the highest ones - in the Sichuan Basin, may indicate the importance of ground forms in aerosols distribution.

**Figure 8:**
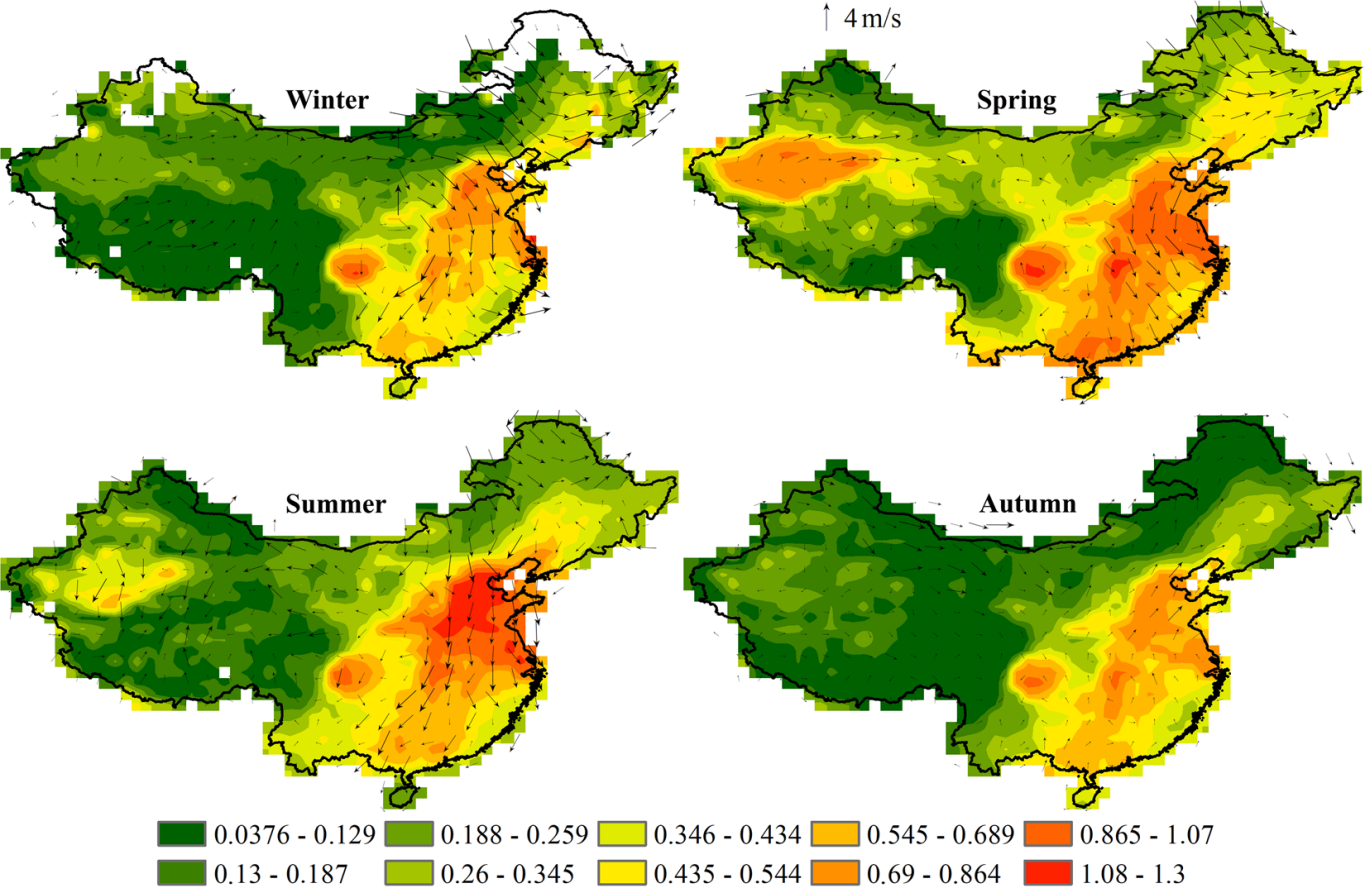
Seasonal mean MODIS AOD from 2000 to 2017 over China. Arrows indicate the Global Data Assimilation System (GDAS) wind vectors at 850hPa.

Seasonal spatial distribution of MODIS AOD over the entire territory of mainland China in the period of 2000–2017 is demonstrated in Fig. 8 and Table 1. High and low annual mean AOD values were observed practically in all regions in summer and winter seasons. Location of centers with high aerosol load was practically not changed during all seasons, but had some seasonal fluctuations, save only the Tarim Basin, where the predominance of dust aerosols with mean AOD values (±standard deviation) 0.592 (±0.176), which are attributed to a great number of dust storms in this period, is prominent in spring^5,13,36,66,77^. AOD values over the North China Plain, the Yangtze River Delta and the Sichuan Basin in spring (0.685 (±0.116), 0.811 (±0.1) and 0.644 (±0.119)) and in summer (0.915 (±0.178), 0.788 (±0.22) and 0.569 (±0.146)) by about 0.2 higher, than in autumn (0.546 (±0.115), 0.613 (±0.093) and 0.443 (±0.124)) and in winter (0.554 (±0.179), 0.695 (±0.115) and 0.422 (±0.156)). Similar seasonal differences in AOD values were also found over Northeast China, but with not so significant fluctuations from spring (0.378 (±0.174)) and summer (0.289 (±0.116)) to autumn (0.195 (±0.086)) and winter (0.233 (±0.13)). Over the Gobi Desert AOD values are insignificantly vary from season to season, with high values in spring (0.257 (±0.06)) and low ones in autumn (0.119 (±0.024)). Over the territory of the Tibetan Plateau AOD values remain within 0.143 (±0.053) during a year with insignificant raise in spring (0.206 (±0.039)), which by 0.1 is higher, than in winter and autumn. Over the territory of the Pearl River Delta the highest seasonal fluctuation of AOD values has been observed with the highest values in spring (0.75 (±0.224)), and the lowest ones in summer (0.453 (±0.14)). Sites without AOD data were registered in winter over Northeast China, which may be caused by a thick mantle of snow in winter and also may increase surface albedo and, consequently, results in failure of MODIS AOD retrieval.

Distribution of MISR AOD (Table 1) also shows very high AOD values in the North China Plain, the Yangtze River Delta, the Sichuan Basin and the Pearl River Delta and low ones over the Tibetan Plateau and the Gobi Desert, demonstrating similar results with a slight underestimation of data against MODIS. Seasonal indicators of the distribution of MISR AOD, also demonstrate similar rolled values with MODIS AOD. Seasonal averages of MISR AOD over China varied with a maximum in spring (0.441 (±0.035)), in summer (0.361 (±0.045)), in winter (0.266 (±0.037)) to a minimum - in autumn (0.259 (±0.038)) with average during the year 0.332 (±0.069). For all regions, with the exception of North China Plain in which the maximum occurs in summer (0.917 (±0.161)) and spring (0.66 (±0.084)) to a minimum - in winter (0.521 (±0.078)) and spring (0.529 (±0.112)), there is a similar seasonal pattern of changes in AOD with maximum values in spring from 0.79 (±0.089) and 0.709 (±0.183) over Yangtze River Delta and Pearl River Delta to 0.267 (±0.038) and 0.199 (±0.045) over the Gobi Desert and the Tibetan Plateau, in summer from 0.765 (±0.159) and 0.541 (±0.139) over the Yangtze River Delta and the Sichuan Basin to 0.216 (±0.037) and 0.129 (±0.032) over the Gobi Desert and the Tibetan Plateau. In general, the Gobi Desert and the Tibetan Plateau regions are less polluted regions of the country with minimum values in the autumn-winter period, the AOD indicators in these regions range from 0.099 (±0.026) to 0.136 (±0.028). In other regions of the country, the autumn-winter period is also characterized by a lower content of aerosols in the atmosphere, so AOD figures above the Sichuan Basin are 0.429 (±0.141) and 0.407 (±0.143), over the Northeast China 0.212 (±0.03) and 0.179 (±0.026). Generally, in regions with low AOD values, where MODIS and MISR AOD values are less than 0.25, the difference between MODIS and MISR AOD is less than 0.02. In regions with medium AOD values (up to 0.65) the difference is commonly as little as 0.03. Subject to high aerosol load with AOD values over 0.65, MODIS values are higher, than that of MISR by more than 0.03.

In order to study meteorology impact on seasonal and monthly AOD variations, we have compared it against surface wind speed (WS, m/s), precipitation (PR, mm) and surface temperature (T, °C) (Table 2). The North China Plain is one of the most polluted region of the country, characterized by gradual increase of AOD values from January (0.554) to August (0.812). With that maximum AOD values were in summer, and minimum ones - in autumn period. Although in summer the amount of precipitation in this region increases, but at high temperature and humidity we observe acceleration of gas transformation into particles and hygroscopic growth of aerosol particles^14,42,64,78^. Also in summer we observe the lowest annual wind speed, which attenuates diffusion processes and, as a consequence, atmosphere-cleaning processes^35,43,74^. At the end of winter and in spring there are frequent sandstorms in north western and northern parts of China, where under the action of air masses dust is carried to all regions of China, indicating that spring increases of AOD values occur predominantly due to dust events.

**Table 2:** Monthly variations of MODIS AOD, wind speed (m/s), precipitation (mm) and temperature (°C) in different regions of China for the period 2000–2017.

	Yangtze River Delta				Pearl River Delta				Tibetan Plateau				Tarim Basin			
	AOD	WS	PR	T	AOD	WS	PR	T	AOD	WS	PR	T	AOD	WS	PR	T
January	0.705	3.1	2.5	4.0	0.488	2.6	36.4	13.3	0.092	5.3	6.6	−12.9	0.164	2.8	4.3	−10.1
February	0.751	3.2	3.2	6.0	0.530	2.5	63.0	15.5	0.138	5.8	9.4	−10.5	0.281	3.1	5.1	−5.6
March	0.785	3.1	7.5	9.9	0.874	2.4	95.4	18.4	0.183	5.3	16.9	−6.9	0.569	3.5	8.6	2.3
April	0.834	2.9	22.2	15.5	0.833	2.2	197.2	22.1	0.235	5.0	24.5	−2.2	0.692	4.0	11.4	9.5
May	0.816	2.7	43.9	20.3	0.516	2.0	310.7	24.8	0.201	4.6	37.6	2.3	0.517	4.0	16.2	14.9
June	1.002	2.4	83.4	23.8	0.414	2.0	358.2	26.5	0.189	4.2	93.6	7.2	0.351	3.8	17.7	19.5
July	0.699	2.5	140.9	27.6	0.407	2.0	249.6	27.3	0.152	3.8	134.6	9.6	0.290	3.7	17.5	21.7
August	0.664	2.6	120.4	26.7	0.519	1.8	287.6	27.4	0.141	3.6	128.0	9.1	0.293	3.6	13.0	20.6
September	0.639	2.6	61.9	22.7	0.560	2.0	197.6	25.9	0.102	3.8	67.8	5.9	0.232	3.4	6.6	15.1
October	0.600	2.7	21.4	17.9	0.572	2.3	76.0	23.1	0.113	3.9	20.3	−1.1	0.158	3.1	5.6	7.3
November	0.601	2.8	9.2	12.2	0.421	2.5	33.9	19.1	0.107	4.4	5.8	−7.1	0.134	2.9	5.4	−0.8
December	0.630	3.0	5.6	6.0	0.429	2.7	22.4	14.3	0.072	4.9	5.4	−10.3	0.114	2.8	4.5	−7.7
	**Sichuan Basin**				**Northeast China**				**North China Plain**				**Gobi Desert**			
	**AOD**	**WS**	**PR**	**T**	**AOD**	**WS**	**PR**	**T**	**AOD**	**WS**	**PR**	**T**	**AOD**	**WS**	**PR**	**T**
January	0.322	2.5	7.6	1.1	0.183	3.2	2.5	−20.6	0.554	3.4	3.9	−3.0	0.147	3.7	0.8	−12.6
February	0.553	2.6	10.1	3.8	0.345	3.3	3.2	−16.5	0.679	3.6	6.5	0.9	0.180	4.0	0.8	−7.7
March	0.661	2.7	25.3	8.5	0.447	3.6	7.5	−7.3	0.653	4.0	11.5	7.3	0.259	4.7	1.7	0.4
April	0.628	2.8	55.3	13.5	0.356	4.0	22.2	4.2	0.691	4.1	24.1	14.1	0.290	5.2	4.1	9.6
May	0.585	2.6	92.2	17.2	0.333	3.7	43.9	13.0	0.713	3.7	35.7	20.1	0.224	5.1	7.1	16.6
June	0.508	2.3	127.6	20.5	0.336	2.9	83.4	19.1	0.942	3.1	70.4	24.7	0.240	4.5	14.2	22.4
July	0.497	2.3	170.0	22.8	0.310	2.8	140.9	21.3	0.993	2.7	168.0	26.0	0.201	4.3	32.1	25.2
August	0.582	2.2	152.9	21.8	0.222	2.8	120.4	19.3	0.812	2.5	141.2	24.1	0.178	4.2	29.8	22.9
September	0.488	2.4	124.3	17.7	0.150	3.0	61.9	12.5	0.642	2.6	54.8	19.6	0.126	4.0	11.4	16.2
October	0.413	2.2	64.6	12.9	0.161	3.5	21.4	3.0	0.534	3.2	24.8	14.0	0.110	4.1	3.9	7.5
November	0.428	2.3	21.0	7.6	0.275	3.4	9.2	−9.1	0.465	3.4	12.0	5.8	0.124	4.2	1.5	−2.5
December	0.306	2.3	8.1	2.5	0.173	3.2	5.6	−18.5	0.429	3.5	4.3	−1.1	0.111	4.0	0.4	−10.2

The Yangtze River Delta is also one of the most polluted regions of the country, where the increase of AOD values occurs with increase of temperature from December to June. The profile of time series of mean AOD values remains high throughout the year (over 0.6). Given that in the middle and low Yangtze River Delta June is the month of grain crops harvesting, straw combustion results in emission of a great amount of aerosols, demonstrating maximum AOD values in this month (1.002)^39,40,43,68^. Also AOD values may be overestimated due to high relative humidity (or moisture vapor) in warm months. However, upon occurrence of Asian summer monsoon we observe a gradual decrease of aerosol load on the region, related with heavy rains, which last up to August, and relatively high wind speed, playing a key role in aerosol diffusion. With this the most intensive diffusion process is seen in July and August. In December Asian winter monsoon carries dry and clean air with minimum AOD values to the region^77^.

In the Sichuan Basin atmospheric precipitations have a great impact on formation of seasonal picture of AOD variations. Seasonal AOD variation is characterized by two distribution peaks - in spring and in autumn with predominance of coarse particles in the total concentration of aerosol particles. In summer finer fraction aerosols prevail. A great amount of aerosol coarse particles in winter and spring, is likely caused by coal combustion and pollution from city and industrial enterprises^5,45^ as well as sandstorms impact^36,53,54,77^. In summer concentration of coarse particles remains at high level until their removal from the atmosphere as a result of heavy precipitations, carried by Asian summer monsoon. In winter their concentration declines due to dry and clean air, carried by Asian winter monsoon^22^. Also in summer months we observe domination of secondary fine particles, formed in the process of photochemical reactions at high temperatures and high humidity. Moreover, straw combustion in the open air is a common practice in all regions of the country. But a great amount of straw is burned incompletely, as a result emanating heavy smoke, consisting of organic particles. This heavy smoke may significantly increase the concentration of fine-size aerosol in the period of crop harvesting from June to September.

Highly concentrated aerosols in the Pearl River Delta, probably, consist mainly of secondary particles, formed from urban and industrial emissions and biomass combustion from various agricultural activity^41,76,78^. In the Pearl River Delta mean AOD value exceeds 0.4 throughout the year, provided that aerosol particles are produced mainly as a result of photochemical reactions in atmosphere and biomass burning. Spring dust storms also affect one of the most distant to the source of their generation regions, demonstrating maximum AOD values in the first two months (March (0.874) and April (0.833)). Also, apparently, the highest aerosol concentration was caused by their generation as a result of direct emissions from various anthropogenic activity and formation of secondary hygroscopic particles at high relative humidity^22,42^. Prominent humid particles removal from atmosphere occurs in the process of cloud scavenging and precipitation scavenging in the period of Asian summer monsoon. The process of aerosol growth through water vapor condensation thereon results in formation of cloud particles, which subsequently fall as precipitations and contribute to removal of aerosol particles from atmosphere^6^. The most minimum AOD values in the Pearl River Delta, just as in all southern regions of China (the Tibetan Plateau, the Sichuan Basin), were in winter during the activity of Asian winter monsoon, which is characterized by clean air and dry weather. With this the lowest wind speed in the Pearl River Delta was with slight seasonal fluctuations.

The Tarim Basin and the Gobi Desert are one of the primary sources of dust not only in China, but in all Asian region. They are regions of arid and semiarid climate with desert and semidesert landscapes, with hot dry summer and cold dry winter. Maximum AOD values in the both regions are observed in spring period, characterized by intensification of dust storms with maximums in April (0.692 and 0.290, respectively). Some of high wind speeds, capable to raise local dust and move it to other regions of the country, are observed in these regions. Due to low amount of precipitations and relatively high wind speeds these regions are subject to soil erosion. Besides, about 90% of all grass fires in China occur in steppe landscapes of the Gobi Desert, resulting in emission of smoke, containing a large amount of black carbon^1,44,63^. Low AOD values from June to October are most likely conditioned by reduction in wind speed and increase an amount of precipitations, which also may lead to decrease of dust particles in the atmosphere of regions.

The most highland and the cleanest study region of the country is the Tibetan Plateau, characterized by low density of population and low anthropogenic activities, which demonstrates the lowest and the most stable AOD values throughout the year, although windy spring weather may cause moderate increase of AODs (with maximum in April (0.235)) due to mineral dust as a result of frequent sandstorms. Taking into account that the Tibetan Plateau is a scarcely populated region and is located away from urbanization and industrialization impact, only coarse continental/dust aerosols are found here^78^. The region is subject to the influence of Asian summer monsoon, which is characterized by the increase in wind speed and precipitation enhancement, meanwhile strong winds contribute to the atmosphere cleaning in the region^52,54^.

Northeast China is the northernmost region of the country, characterized by thick natural vegetation with dry cold winter and rainy warm summer. Meanwhile high AOD values in this region were in winter (with maximum in February (0.345)) due to a great amount of solid particles, generated as a result of fossil fuel combustion for heating, forming smoke and soot aerosols. Gradual increase of snow mantle on the ground restricts emission of coarse particles into atmosphere due to erosion reduction. This suggests that principal pollutants are secondary aerosols. Spring dust events and local soil dust emission contribute to increase of aerosol concentration.

## Conclusions

This study with use of Level 2/3 aerosol data sets, obtained from satellite sensors MODIS and MISR, analyzed time-space distribution and trends of aerosol load over different ecological and geographical regions of China in the period from 2000 to 2017, and validation of the data obtained with a ground-based network AERONET was also carried out. Generally, there is a tendency towards gradual decline in aerosol concentration in ecological regions and generally in the country to −0.004 per decade (from 0.351 in 2000 to 0.268 in 2017) for MODIS and to −0.009 (from 0.341 in 2000 to 0.238 in 2017) for MISR, where 18-years AOD values decreased by 23.6% and 30.1% on the average in the whole country, respectively. AOD data, obtained from two spectral radiometers, demonstrates considerable positive correlation relationships (r = 0.747), and comparison of the results obtained over different regions and various underlying terrains with AERONET data demonstrate high relation (*r* = 0.869 − 0.905), while over 60% of the entire sampling fall within the range of the expected tolerance, established by MODIS and MISR over earth (±0.05 ± 0.15 × AOD_AERONET_ and 0.05 ± 0.2 × AOD_AERONET_). There was an expressed overestimate of search results from MODIS by 14–17% and an understatement of 8–22% for MISR, with the best results obtained over the SACOL station with a slight understating of results by 3% for MODIS and 8% for MISR.

During the entire study period (2000–2017) a regional distribution of MODIS AOD with gradual decrease from the east to the west of the country with the highest values in the North China Plain (0.675 (±0.211)), the Yangtze River Delta (0.727 (±0.161)), the Pearl River Delta (0.546 (±0.195)), the Sichuan Basin (0.601 (±0.162)) and the lowest ones in the Tibetan Plateau (0.143 (±0.053)) was clearly identified. Population, geography and relief, climate and economy are closely related to aerosol load of the territory.

Seasonal AOD variation over the whole study territory demonstrated clear annual course with maximums in spring and summer and minimum in autumn and winter. High AOD values are attributed to hygroscopic growth of aerosols, formation of secondary aerosols and pollutants as a result of agricultural biomass combustion after crop harvesting in the adjacent districts, which entails pollutants accumulation in this region. In spring the whole territory of the country is exposed to dust, which comes from the territories of North and Northwest China, leading to increase of AOD. In summer we observe aerosol scavenging as a result of activity of Asian summer rainy monsoon, which results in AOD decrease.

Generally, we observe a gradual decrease of aerosol load on the territory of the country, which may be associated with a consistent state policy in the field of environment protection, aimed not only at control and development of methods to decrease atmosphere pollution, but the improvement of the total ecological situation of the country. MODIS coupled with AERONET has enormous potential to ensure complex evaluation of global aerosol distribution, which may result in reduction of uncertainties with respect to quantitative role of aerosols and their impact on the territory. A continuous extension of monitoring networks of AERONET stations is required to solve these issues. Further studies should be aimed at improvement of identification and investigation of quantitative estimation of natural and anthropogenic activity contribution into the total aerosol volume.
