# Author Correction: Combined use of satellite and surface observations to study aerosol optical depth in different regions of China

**DOI:** 10.1038/s41598-019-54734-6

**Published:** 2019-12-04

**Authors:** Mikalai Filonchyk, Haowen Yan, Zhongrong Zhang, Shuwen Yang, Wei Li, Yanming Li

**Affiliations:** 1 Faculty of Geomatics, Lanzhou Jiaotong University, Lanzhou, 730070 China; 2 Gansu Provincial Engineering Laboratory for National Geographic State Monitoring, Lanzhou, 730070 China; 3 School of Mathematics and Physics, Lanzhou Jiaotong University, Lanzhou, 730070 China; 4 Lanzhou Petrochemical Polytechnic Information Technology and Education Center, Lanzhou, 730070 China

Correction to: *Scientific Reports* 10.1038/s41598-019-42466-6, published online 16 April 2019

This Article contains a typographical error in the Introduction where,

“Also, to study regional and general prerequisites of atmosphere pollution, aerosol properties in many large cities of the country, including Shanghai^46^, Nanjing^47^, Beijing^48^, Guangzhou^49^, Lanzhou^37^, Tianjin^50^ and Xian^51^ have been studied.”

should read:

“Also, to study regional and general prerequisites of atmosphere pollution, aerosol properties in many large cities of the country, including Shanghai^46^, Nanjing^47^, Beijing^48^, Guangzhou^49^, Lanzhou^37^, Tianjin^50^ and Xi’an^51^ have been studied.”

Additionally, in Figures 5 and 6, the numbers on the axes contained commas rather than decimal points. The correct Figures 5 and 6 appear below as Figures 1 and 2.

**Figure 1:**
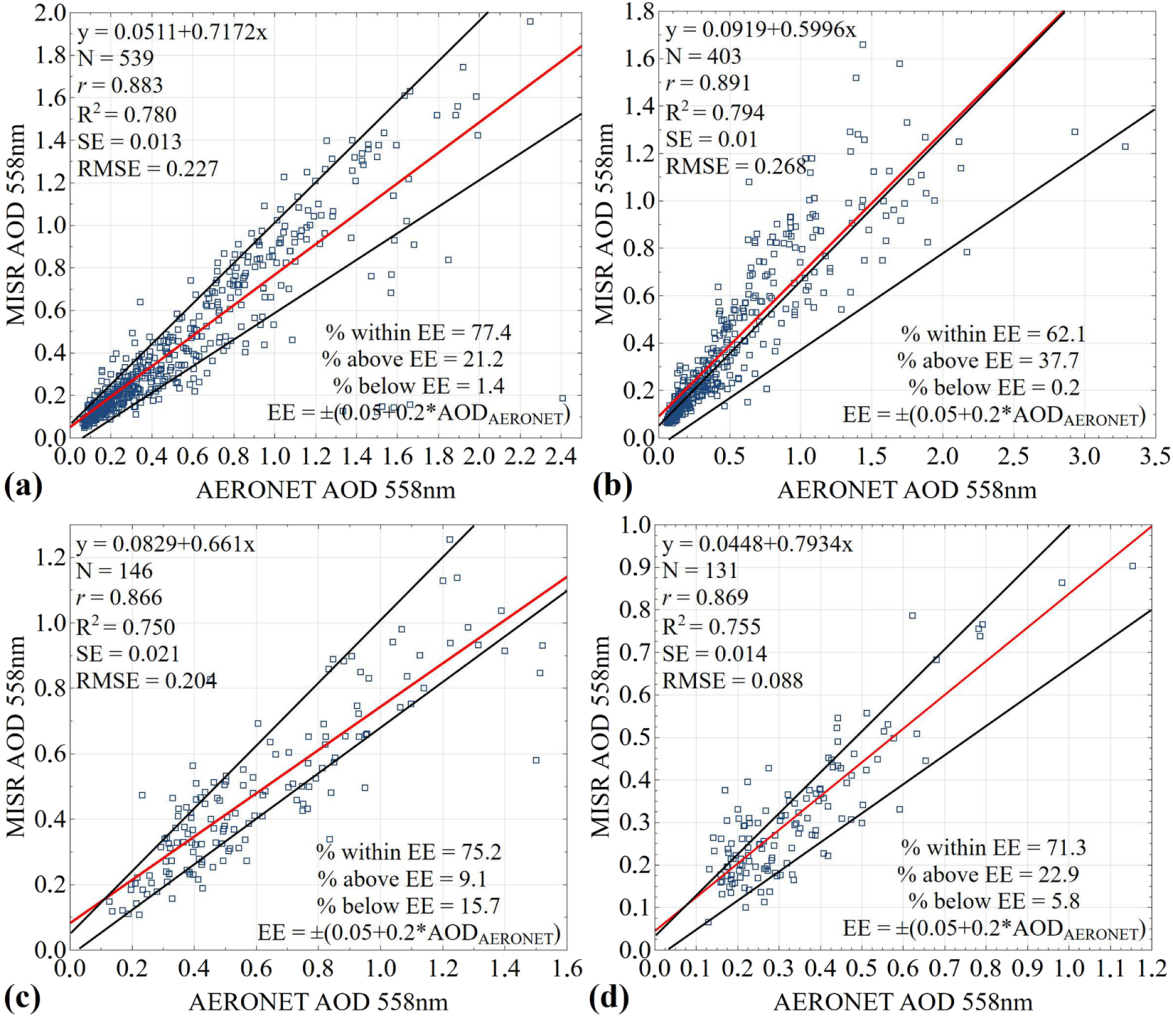
.

**Figure 2:**
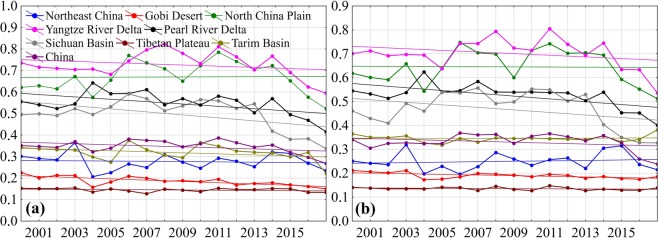
.

